# Dictator Game Giving: The Importance of Descriptive versus Injunctive Norms

**DOI:** 10.1371/journal.pone.0113826

**Published:** 2014-12-10

**Authors:** Nichola J. Raihani, Katherine McAuliffe

**Affiliations:** 1 Department of Genetics, Evolution and Environment, University College London, Gower Street, London, WC1E 6BT United Kingdom; 2 Department of Psychology, Yale University, New Haven, Connecticut, United States of America; Mälardalen University, Sweden

## Abstract

Human behaviour is influenced by social norms but norms can entail two types of information. Descriptive norms refer to what others do in this context, while injunctive norms refer to what ought to be done to ensure social approval. In many real-world situations these norms are often presented concurrently meaning that their independent effects on behaviour are difficult to establish. Here we used an online Dictator Game to test how descriptive and injunctive norms would influence dictator donations when presented independently of one another. In addition, we varied the cost of complying with the norm: By stating that $0.20 or $0.50 cent donations from a $1 stake were normal or suggested, respectively. Specifying a higher target amount was associated with increased mean donation size. In contrast to previous studies, descriptive norms did not seem to influence giving behaviour in this context, whereas injunctive norms were associated with increased likelihood to give at least the target amount to the partner. This raises the question of whether injunctive norms might be more effective than descriptive norms at promoting prosocial behaviour in other settings.

## Introduction

It is well documented that human behaviour deviates consistently from predictions based on economically-rational, agents. One domain in which the departure from expected behaviour is particularly pronounced is in social interactions with other individuals. A slew of laboratory and field studies have shown that people are often more helpful than would be predicted based on short-term, income-maximising strategies (see [1] for a review). Despite this fact, humans are not uniformly prosocial. Instead, considerable variation exists both in the extent to which individuals are willing to help others and the contexts in which they are willing to help (e.g. [2], [3]). Explaining the mechanisms underpinning such variation is now a key goal for researchers interested in the evolution of social behaviour in humans.

One factor underlying how individuals are expected to behave in a given context is how others behave in that setting. The tendency to copy the behaviour of others is widespread in the animal kingdom and might reduce the costs of gathering and processing information required to behave optimally in a given environment [4]–[9]. For example, fish copy the flight responses exhibited by the other members of the shoal without having to take time to assess the type and location of the threat (e.g. [10]). Humans are thought to be especially likely to conform to the behaviour of others in social settings [11]. For example, if asked to give to a charity, an individual may be willing to donate but unsure how much to give. Giving too little risks harming the donor’s reputation whereas giving too much carries unnecessary costs. In situations such as these, where the appropriate behaviour is not always apparent, individuals may often observe how others behave in a similar situation and then copy their behaviour [4]–[9]; [12], [13]. The power of so-called descriptive social norms - information about how others typically behave in that setting - [14] has been demonstrated in a variety of settings. For example, individuals are less likely to drop litter in a clean area than in an already littered area because a clean area suggests that most people do not drop litter; while a littered area suggests otherwise [15]. Similarly, guests who are asked to re-use hotel towels are significantly more likely to do so if they are given additional information stating that other guests who stayed in that room also re-used their towels [16].

In other situations, the appropriate social behaviour may be made apparent by the use of injunctive norms. Whereas descriptive norms give individuals information about what *is* done in a given situation, injunctive norms give information about what *ought* to be done [14]. Implicit in the idea of injunctive norms is the fact that behaviours which violate the code of conduct will be met with moral or social disapproval [14], [17]. In many real-world situations, individuals observe both descriptive and injunctive norms simultaneously [18]. Where these norms are in conflict with one another, experimental evidence has demonstrated that people’s behaviour will vary according to which of the norms they focus on [14]. For example, people were more likely to drop litter after seeing a confederate dropping litter in a littered but unswept area than when seeing the confederate drop litter in a littered but swept area [14]. It was argued that seeing a lot of litter, but swept into a pile made the descriptive and injunctive norms incongruent: many people dropped litter, but littering was disapproved of. Seeing a confederate drop litter in this scenario thus focussed people’s attention on the injunctive norm and thus made them less likely to drop their own litter [14]. Similarly, a study conducted in an endangered forest in the US showed that visitors were more likely to steal pieces of petrified wood (despite visible signs prohibiting such behaviour) if other signs with a contradictory descriptive norm stated “Many past visitors have removed the petrified wood from the park, changing the state of the Petrified Forest” [19]. In contrast, in a large-scale real world experiment conducted on energy use in 287 households in the USA, participants were provided with information about their energy use relative to that of neighbours. For households who were consuming more energy than average, the descriptive norm was successful at reducing energy consumption; however households that were using less energy than average demonstrated a boomerang effect whereby they began to use more energy - thereby complying with the descriptive norm of behaviour. This negative boomerang effect could be removed, however, by adding injunctive information in the form of a smiling or sad emoticon next to the information about the householder’s energy use [20].

Although several studies have tested the relative impact of descriptive and injunctive norms when presented together, fewer studies have asked which type of norm has the largest effect on behaviour. In order to assess the independent effects of descriptive and injunctive norms on behaviour they have to be presented in isolation. Some studies attempting to assess the independent influence of descriptive versus injunctive norms have been conducted in the health sphere and have shown that descriptive norms are often more effective than injunctive norms at eliciting the desired behaviour (e.g. [21], [22] but see [23]). For example, in a study designed to promote healthy eating in adolescents, fruit consumption was increased when subjects were told about how much fruit their peers consumed (descriptive norm). However, when told how much fruit their peers thought they ought to eat (injunctive norm), adolescents did not eat more fruit than under a control condition and actually reported lower fruit take intentions than the control group [22]. This finding hints that descriptive norms may be more effective at promoting behaviour change (at least in the health sphere) and also that injunctive norms may sometimes have a counter-productive effect on behaviour or intentions. Some contrasting results come from a study conducted on retirement saving decisions [24]. Participants in the study were told either that most employees contributed to a retirement saving plan or that an expert advised them to contribute to a retirement saving plan. In this setting, people said they would save more of their income in the injunctive norm treatment than in the descriptive norm treatment.

In the context of social behaviour, a recent study attempted to disentangle the influence of descriptive and injunctive norms on behaviour in the Dictator Game [25]. The Dictator Game is a two-player game where one player, the dictator, is given control of a sum of money and can choose how much of the endowment to share with the partner, the receiver [1], [26]. This game is a useful tool for exploring how norms affect social behaviour since it measures variation in voluntary donations in a one-shot, non-strategic interaction. In their study, Bicchieri & Xiao [25] either gave players a descriptive norm, an injunctive norm, or both. The descriptive norms stated (i) that most other players were fair (gave at least 40% of the endowment to the receiver) or (ii) that most other players were selfish (gave 20% or less of the endowment to the receiver). The injunctive norms used the same reference points but instead of emphasising what other players did, the information given was framed in terms of how other dictators in the game thought the endowment should be divided. Finally, some players were presented with information where the injunctive norm and descriptive norm contradicted one another. The results from the study were somewhat inconclusive. As expected, more players split the endowment fairly with the receiver in the treatments emphasising that fair behaviour was either common (descriptive norm) or expected (injunctive norm) than in the treatments emphasising that selfish behaviour was either common or expected. However, there was no difference in the percentage of players splitting the endowment fairly with the receiver in either of the contradictory information treatments (fair behaviour expected but selfish behaviour common versus fair behaviour common but selfish behaviour expected), which implies that both types of norm influenced behaviour to the same extent [25]. Further analysis, which incorporated subjects’ actual beliefs about descriptive and injunctive norms rather than the exogenous information, suggested that individuals were more likely to split the money fairly when they believed others would also do the same. Beliefs about the expectations of others, on the other hand, did not affect the tendency to share the money equally [25].

While the study above provides a useful starting point for investigating the influence of descriptive versus injunctive norms on prosocial behaviour, there are still many gaps in our understanding. First, the study above did not compare behaviour compared to a baseline where no norm information was given to subjects. Thus, we do not know the extent to which norm information of each type influenced behaviour relative to a neutral control. Second, the study above only measured whether dictators split the endowment fairly with the receiver or not, rather than norm compliance. Crucially, individuals who were given the ‘selfish’ norm information could still have complied with the norm by giving the ‘selfish’ amount but this was not measured. Thus, it is not known from this study whether norm compliance was higher when complying with the norm was relatively cheap versus when it was relatively costly. Finally, it is possible that specifying a high level of prosocial behaviour might have the perverse effect of making individuals less likely to comply. Such an effect might arise because individuals who are unwilling to bear the costs associated with the suggested level of giving might not experience a warm glow of giving [27] or a positive self-image if they give less than is suggested. Under these conditions, individuals might be more likely succumb to the so-called ‘what the hell’ effect [28] (pp 127–131) where a completely selfish strategy (i.e. keeping the entire endowment in a Dictator Game) yields greater utility than giving away an amount that is too low to be reconciled with a positive self-image. In the previous study investigating the effect of norms on Dictator Game giving [25], dictators were not allowed to keep the entire endowment meaning that this ‘what the hell’ effect could not be investigated.

Here we used the Dictator Game, to test the impact of descriptive versus injunctive norms on voluntary donations. A previous metastudy of Dictator Games has shown that dictators typically transfer around 28% of the stake to receivers [29]. We asked whether normative information could induce dictators to give either (i) 20% of their stake or (ii) 50% of their stake to receivers; and whether the type of norm (descriptive versus injunctive) affected dictator compliance. A better understanding of the applications and limitations of descriptive and injunctive norms to influence prosocial behaviour has important applied value. For example, museums typically rely on donations from visitors to pay for overheads but it is not clear whether suggested donations (injunctive norms) or descriptive information would be more effective at increasing either the probability of receiving a donation or increasing the size of the donation. While previous studies have shown that normative information can be useful to encourage prosocial behaviour in such settings, it is important to know (i) whether descriptive or injunctive norms are likely to be more effective and (ii) whether emphasising a very costly target behaviour may have the unintended consequence of making people behave even more selfishly.

## Methods

This project was approved by the University College London ethics board under the project number 3720/001. All data were collected in August 2014 using the online labour market, Amazon Mechanical Turk (www.mturk.com). We recruited workers from the USA to play in an online Dictator Games (described below and see supplementary materials for instructions given to players). Of the 2,400 workers recruited to play the game, 1,200 were randomly assigned to the active role of ‘dictator’ and the remaining 1,200 to the passive role of ‘receiver’ (though the more neutral terms ‘Player 1’ and ‘Player 2’ were used in the information seen by subjects and are used hereafter). Since only Player 1 is active in this game, all analyses were restricted to data from Player 1 only. Prior to taking part in the Dictator Game, subjects were first asked to provide some background demographic information on their age, gender, education and income levels (see S1 Table in S1 File; data shown for individuals allocated to Player 1 role only). Some individuals (n = 29 of those allocated to Player 1 role) did not supply the required demographic information on age and gender. Where this information was included in analyses, the data from these individuals were excluded. Data from a further 18 individuals allocated to the role of Player 1 were excluded, either because individuals took part in the task more than once or because they did not complete the task. As a consequence, sample sizes for analyses deviated slightly from the number of subjects who were recruited to play the game. No deception was used in this study and participants were not debriefed as to the purpose of the study after the game. Player 1 was told that they were allowed to choose how a $1 stake would be distributed between themselves and Player 2. Although this stake size is relatively small compared to studies conducted under laboratory settings, a recent study conducted using Amazon Mechanical Turk (MTurk) workers found no difference in dictator behaviour based on stake sizes of $1, $5 or $10 (when dictators were recruited from the USA [30]). Players were matched with partners ex-post (as in [30]). MTurk workers are identified by a unique 14-digit code rather than their names. Workers were told that their ID would not be revealed to their partner in the game, thus ensuring anonymity.

The 1,200 individuals assigned to the role of Player 1 were randomly assigned to one of six treatments (n = 200 individuals per treatment but sample sizes available for analyses shown in parentheses below). The treatments varied with respect to the information that Player 1 received prior to playing the game (see below). In all treatments, Player 1 was informed that Player 2 would see the information and was required to answer two comprehension question correctly to indicate that they understood the rules of the game. Players who answered either of these questions incorrectly were not permitted to take part in the experiment.

Treatment 1: Most Player 1s give $0.20 or more to Player 2 (n = 198).

Treatment 2: Most Player 1s give $0.50 or more to Player 2 (n = 200).

Treatment 3: It is suggested that Player 1 give $0.20 or more to Player 2 (n = 197).

Treatment 4: It is suggested that Player 1 give $0.50 or more to Player 2 (n = 196).

Treatment 5 (control 1): Most people on MTurk are at least 20 years old (n = 192).

Treatment 6 (control 2): Most people on MTurk are less than 50 years old (n = 199).

Treatment 1 and 2 were designed to test whether a descriptive norm about behaviour in the game would affect Player 1′s behaviour, and whether the cost involved in adhering to the norm ($0.20 versus $0.50) affected whether Player 1 would conform to the norm. Treatments 3 and 4 were designed to test whether injunctive norms or descriptive norms, respectively, were more effective at influencing Player 1′s behaviour. Treatments 5 and 6 were included as control treatments. We included the number ‘20’ in treatment 5 and the number ‘50’ in treatment 6 to control for the possibility that these numbers might have acted as an anchor [31] for donation amounts in the other treatments. Anchoring occurs when the presentation of the first piece of numerical information influences judgements and decisions made thereafter [32]. For example, an arbitrary number rolled on a ‘wheel of fortune’ has been shown to influence guesses about the percentage of African countries in the United Nations: people who were exposed to a high arbitrary anchor guessed higher percentages than those exposed to a lower anchor [31].

## Analysis

We first asked whether descriptive or injunctive norms, respectively, were associated with increased compliance relative to a neutral control. We coded the propensity to comply with the norm information as ‘1’ if Player 1 gave at least the target amount ($0.20 or $0.50, respectively) to Player 2 and ‘0’ if Player 1 gave less than the target amount to Player 2. We analysed data using two generalized linear models (GLM) with binomial error structure. Separate models for the $0.20 target amount and the $0.50 target amount were produced. For each analysis, a global model was specified which included the following terms: age, gender and treatment (‘descriptive’, ‘injunctive’, ‘control’). For players in the control conditions, we specified the response as ‘1’ if the player gave at least the target amount specified by the matched norm amount (i.e. if in the ‘age 20’ control, player donation set as ‘1’ if it exceeds $0.20 or more), and ‘0’ otherwise. Thus, we can compare how many players give at least $0.20 or $0.50 (respectively) in the norm treatments compared to players in the matched control conditions.

Next, we asked whether mean donations varied according to the norm information (‘descriptive’, ‘injunctive’) or the target amount specified (‘$0.20’, ‘$0.50’). Control data were not included in this analysis as no target amount was specified in the control treatments. The amount given by Player 1 (‘donation’) was set as the response term in a linear model (LM) with Gaussian error structure. The global model included the terms age, gender, target amount, treatment and the 2-way interaction between treatment and amount.

Finally, we tested the idea that specifying a large target donation ($0.50 versus $0.20) might have the perverse effect of making Player 1 more likely to give nothing at all to Player 2. If Player 1 gave nothing to Player 2 we coded this as ‘1’ and if Player 1 gave a non-zero donation to Player 2 then this was coded as ‘0’. Data were analysed using a GLM with binomial error structure, with the following explanatory terms: age, gender, treatment (‘descriptive’/‘injunctive’), target amount (‘$0.20’/‘$0.50’), and the 2-way interaction between treatment and amount.

All data were analysed in the statistical package, R [33]. We used an information theoretic approach with model averaging as described in [34]. Under an information-theoretic approach, a series of candidate models are generated, with each model representing an hypothesis. Rather than testing a null hypothesis, the relative degree of support for each model from the candidate set is calculated [35]. By comparing different models, it is possible to determine the relative importance of different explanatory terms. Following the specification of the global model, the input variables were standardized according to [36]. Standardizing input variables allows the relative strength of parameter estimates to be interpreted. We used the package MuMIn [37] to derive and compare submodels from this initial global model. Models were compared to one another using Akaike’s Information Criterion corrected for small sample sizes (AICc) [38]. Following specification of the global mode, a subset of ‘top models’ were defined by taking the best model (the model with the lowest AICc value) and any models within 2AICc units of the best model (following [35]). Using this subset of models, we computed the average parameter estimates for each term included in the subset of models, as well as the relative importance of the term. Importance is calculated by summing the Akaike weights of all models where the term in question is included in the model. Akaike weights represent the probability of a given model being the true model (compared to other candidate models in the set) [35]. Importance can therefore be thought of as the probability that the term in question is a component of the best model [39]. Confidence intervals associated with parameter estimates indicate how accurate the estimates are likely to be: confidence intervals that span zero indicate that there is little evidence that the predictor variable affects the response term [34]. In the results section, we only present the parameter estimates from the top models (those that were within 2 AICc units of the best model). All data and R code are available as supplementary materials (S1 File and S2 File).

## Results

**Figure 1:**
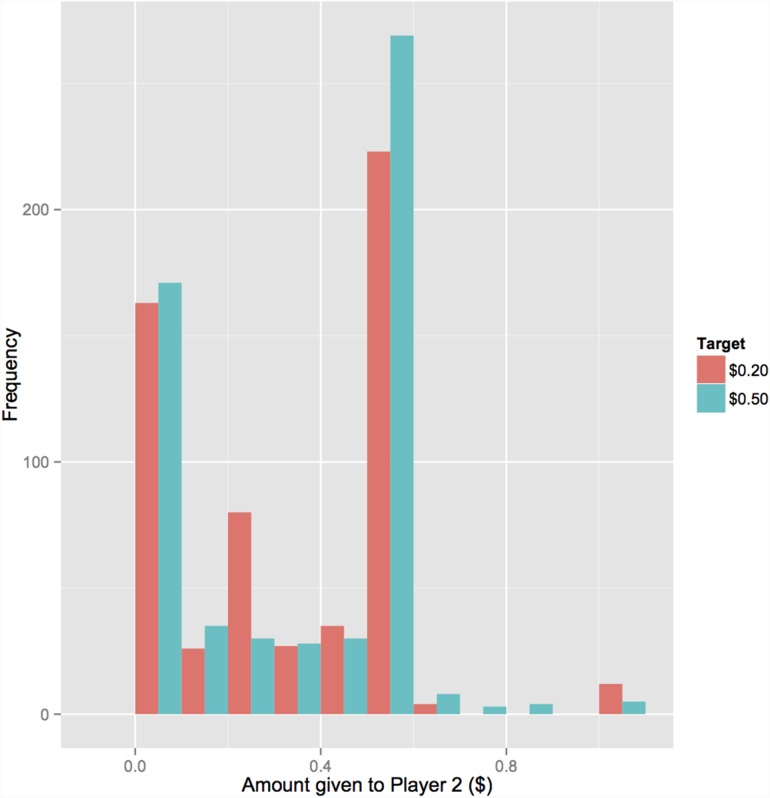
Histogram of donations Player 1 gave to Player 2 across Experiment 1 and Experiment 2. Data from control treatments are not shown. Red columns are data from players who saw the $0.20 target amount; blue columns are data from players who saw the $0.50 target amount.

The most common donation across all conditions (excluding controls) was $0.50 (Fig. 1). Pooling data across norm types, players were most likely to give $0.50 or more in the condition where the target of $0.50 was emphasised in the norm information (220/396, 55.6%) than in the condition where the target of $0.20 or more was emphasised in the norm information (157/395, 39.7%; Chi-squared test: χ^2^ = 19.2, df = 1, p<0.001). This was apparently not an artefact of a larger number acting as an anchor in the $0.50 target condition since we found that players were slightly more likely to give $0.50 or more in the ‘age 20’ control condition (93/192, 48.4%) than in the ‘age 50’ control condition (75/199, 37.7%), which is the opposite of what we would have expected if the number 50 was anchoring players towards making larger donations (Chi-squared test: χ^2^ = 4.18, df = 1, p = 0.04). As expected, compliance was reduced when a higher target amount was specified. Players were less likely to give a target amount of at least $0.50 (220/396; 55.6% complied) than a target amount of at least $0.20 (273/395; 69.1% complied; Chi-squared test: χ^2^ = 14.9, df = 1, p = 0.0001).

**Figure 2:**
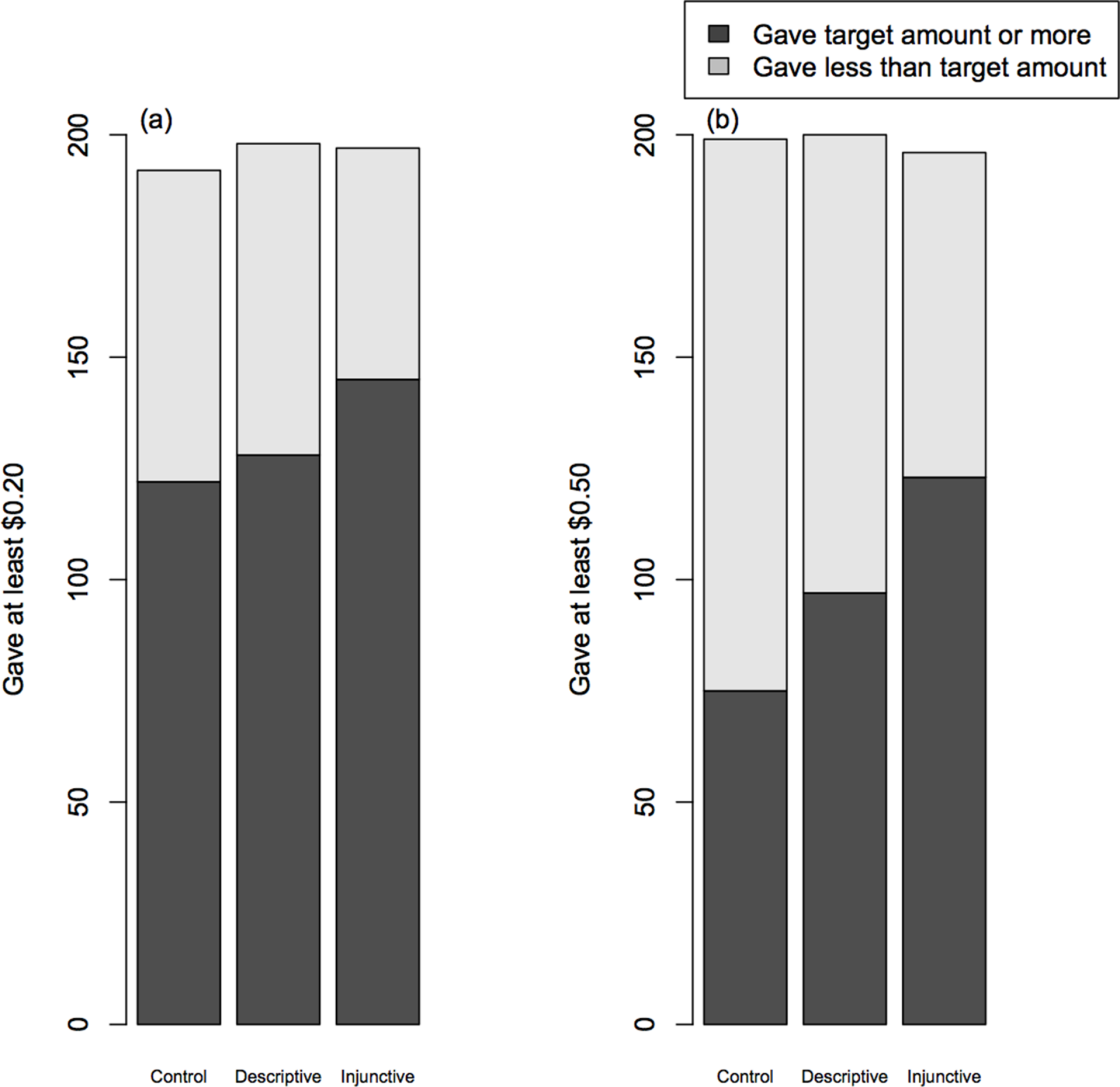
Numbers of Player 1 who complied with the norm to give (a) at least $0.20 or (b) at least $0.50 to Player 2 according to the type of norm information that was used in the instructions. Control data are those where no norm information was shown.

Injunctive norms were associated with increased compliance relative to controls, both when the target amount was $0.20 (effect size: 0.55±0.23; Table 1) and when the target amount was $0.50 (effect size: 0.96±0.21; Tables 2 & 3; Fig. 2). By contrast, relative to controls, there was no evidence that descriptive norms increased compliance when the target amount was $0.20 (effect size: 0.09±0.22; Table 1). The effect of descriptive norms (relative to controls) seemed to be slightly stronger in the ‘give $0.50’ condition (effect size: 0.35±0.21) but the confidence intervals for this effect still spanned zero (Table 3; Fig. 2). Males were less likely than females to comply with the ‘give $0.20’ norm but this gender effect was not replicated in the ‘give $0.50’ condition. Similarly, we found a positive effect of age on tendency to comply with the ‘give $0.20’ norm (Table 1) but the effect was only marginal in the ‘give $0.50’ condition (Table 3). Mean donations increased when a higher target amount was specified (Tables 4 & 5) regardless of whether the target was specified via a descriptive or an injunctive norm. Supporting the previous analyses, men tended to make smaller mean donations than women and mean donation size increased with age (Tables 4 & 5).

**Table 1:** GLM to investigate factors affecting probability that Player 1 would comply with the ‘give $0.20’ norm.

Parameter	Estimate	SE	Confidence Interval
Intercept	0.52	0.15	(0.22, 0.82)
Age	0.55	0.20	(0.16, 0.96)
Gender (male)	–0.41	0.19	(–0.78, −0.05)
Treatment			
Descriptive	0.09	0.22	(–0.33, 0.52)
Injunctive	0.55	0.23	(0.11, 1.00)

Only one top model was supplied so estimates, standard errors and confidence intervals for all terms in the model are shown below. For treatment, ‘control’ was set as the reference category. For gender, ‘female’ was set as the reference category.

**Table 2:** GLM to investigate factors affecting probability that Player 1 would comply with the ‘give $0.50’ norm.

Model Rank	Parameters	df	AICc	*w^i^*
**1**	**Age + Treatment**	**4**	**783.7**	**0.32**
2	Treatment	3	784.1	0.26
3	Gender + Treatment	4	784.5	0.21
4	Age + Gender + Treatment	5	784.6	0.21

The table shows the top models (models within 2AICc units of the best model), with AICc values and Akaike weights (*w^i^*). The best model is highlighted.

**Table 3:** Estimates, standard errors and confidence intervals for parameters included in the top models investigating factors affecting compliance with the ‘give $0.50’ norm.

Parameter	Estimate	SE	Confidence Interval	Importance
Intercept	–0.44	0.15	(–0.74, −0.15)	
Treatment				1.00
Descriptive	0.35	0.21	(–0.06, 0.76)	
Injunctive	0.96	0.21	(0.55, 1.37)	
Age	0.26	0.17	(–0.08, 0.60)	0.53
Gender (male)	–0.20	0.17	(–0.54, 0.14)	0.42

Effect sizes have been standardized on two SD following [36]. Standard errors are unconditional, meaning that they incorporate model selection uncertainty [34].

**Table 4:** GLM to investigate factors affecting mean donation made by Player 1.

Model Rank	Parameters	df	AICc	*w^i^*
**1**	**Target + Gender + Treatment +** **Age + Target: Treatment**	**7**	**–58.7**	**0.39**
2	Target + Gender + Treatment + Age	6	–58.2	0.30
3	Target + Gender + Age	5	–57.0	0.17
4	Target + Treatment + Age + Target: Treatment	6	–56.7	0.14

The table shows the top models (models within 2AICc units of the best model), with AICc values and Akaike weights (*w^i^*). The best model is highlighted.

**Table 5:** Estimates, unconditional standard errors, confidence intervals and relative importance for parameters included in the top models for Table 4.

Parameter	Estimate	Unconditional SE	Confidence Interval	Importance
Intercept	0.31	0.01	(0.29, 0.33)	
Target	0.00	0.00	(0.00, 0.003)	1.00
Age	0.04	0.02	(0.00, 0.07)	1
Gender (female)	–0.03	0.02	(–0.07, −0.00)	0.86
Treatment (injunctive)	0.03	0.02	(–0.00, 0.06)	0.83
Target: Treatment	0.00	0.00	(–0.00, 0.00)	0.53

Effect sizes have been standardized on two SD following [36]. Standard errors are unconditional, meaning that they incorporate model selection uncertainty [34].

Finally, we asked whether specifying a large target amount ($0.50 as opposed to $0.20) might have the perverse effect of making players more likely to give nothing at all. Of the 1,182 subjects available for analysis, 341 (28.8%) gave none of the endowment to Player 2. The results from the GLM indicated that males were more likely than females to give none of the endowment to Player 2; and there was a small negative effect of age on propensity to keep the entire endowment (Tables 6 & 7). The type of norm (descriptive versus injunctive) did not seem to affect whether Player 1 kept the entire endowment and, contrary to the prediction based on the ‘what the hell’ hypothesis, there was no discernible effect of target amount on propensity to keep the entire endowment (Table 7).

**Table 6:** GLM to investigate factors affecting probability that Player 1 would give nothing to Player 2.

Model Rank	Parameters	df	AICc	*w^i^*
**1**	**Gender + Age**	**3**	**878.2**	**0.47**
2	Gender + Treatment + Age	4	878.9	0.34
3	Gender	2	880	0.20

The table shows the top models (models within 2AICc units of the best model), with AICc values and Akaike weights (*w^i^*). The best model is highlighted.

**Table 7:** Estimates, unconditional standard errors, confidence intervals and relative importance for parameters included in the top models for Table 6.

Parameter	Estimate	Unconditional SE	Confidence Interval	Importance
Intercept	–1.04	0.08	(–1.20, −0.88)	
Gender	0.48	0.17	(0.15, 0.82)	1.00
Age	–0.34	0.18	(–0.70, 0.02)	0.80
Treatment (injunctive)	–0.19	0.17	(–0.52, 0.13)	0.34

Effect sizes have been standardized on two SD following [36]. Standard errors are unconditional, meaning that they incorporate model selection uncertainty [34].

## Discussion

We have shown that donations in an online Dictator Game are influenced by injunctive but not descriptive norms. In contrast to a previous study investigating the effect of norms on Dictator Game giving [25], here we found that injunctive norms were slightly more effective than descriptive norms at eliciting compliance. This agrees with findings from a previous paper where participants’ beliefs about others’ expectations had a greater influence on dictator game giving than participants’ beliefs about others’ behaviour [40]. Although we observed less compliance in the ‘give $0.50’ condition than in the ‘give $0.20’ condition, mean donations did increase when subjects were given the $0.50 target amount compared to the $0.20 target amount, indicating that specifying higher target amounts might result in higher mean donations even if many people give less than the amount specified.

People are exposed to both injunctive and descriptive norms of behaviour in several real-world settings. Often, however, these different types of norm may be presented together and may either be congruent or might contradict one another [18] which makes it difficult to know when descriptive or injunctive norms, respectively, might exert a greater influence on behaviour. Although several previous studies have highlighted descriptive norms as being salient drivers of behaviour (e.g. [14], [16], [21]), here we found that an injunctive norm was more effective at encouraging people to comply with the norm of either giving at least $0.20 or giving at least $0.50 to the partner in a Dictator Game. This effect occurred despite the injunctive norm being phrased as a relatively weak ‘it is suggested that’, as opposed to an arguably more forceful formulation, such as ‘you ought to’ or ‘you should’. Injunctive norms may have been particularly effective because, in this experiment, the injunctive norm was apparently given by the experimenter, who might be perceived as a legitimate authority to be obeyed (c.f. [41]). In the previous study investigating Dictator Game giving, information about what level of sharing was expected apparently came from other dictators who might not have been perceived as authorities to be obeyed [25]. It might also be the case that individuals are more likely to comply with injunctive norms if the individual who requests compliance does not stand to benefit from the behaviour. For example, in our Dictator Game, the experimenter did not stand to benefit if Player 1 complied with the injunctive norm. Conversely, in several real-world situations, the likely beneficiary of the prosocial behaviour is also the person who gives the injunctive norm. For example, museums often suggest a donation amount from visitors but in this case it is the museum that will receive the donation that the visitor makes. It would be interesting to explore whether injunctive norms that apparently reflect Player 2′s opinion (e.g. ‘Player 2 suggests that Player 1 give $0.50 or more’) would also elicit similar behaviour from Player 1. The efficacy of normative feedback has also been shown to vary with other factors, such as political ideology. For example, descriptive and injunctive feedback about household energy use prompted greater reductions in energy consumption in households with politically liberal ideology than conservative households [42]. We did not ask players about their political ideology meaning that we could not test how this and other factors might have influenced compliance with descriptive and injunctive norms, respectively. This remains a possible avenue for further investigation.

It may be the case that we found lower compliance for descriptive versus injunctive norms in this setting because people simply did not believe the descriptive norm information. Subject belief is important in this study - unlike most real world settings where subjects can directly observe how others behave in that situation. By contrast, since our injunctive norm entailed an instruction rather than information, compliance should not be affected by subject beliefs. We did not include a question in this study to ask subjects whether they believed the descriptive norm information, though previous data indicate that subjects do typically trust that the game instructions are accurate and that they are not deceived (Raihani & Bshary, in review). Moreover, we did not use deception in this or any previous studies, meaning that our Requester ID (the name under which experiments are posted) is not known for deceiving participants. Finally, the amounts we specified in the norm information are consistent with patterns observed in previous Dictator Games in this setting (e.g. [43]): most people do give half of the endowment to the partner and this figure may therefore be plausible to most players. We therefore have no reason to suspect that subjects selectively disbelieved the descriptive norm information.

Previous work has argued that descriptive and injunctive norms are concerned with fundamentally different goals, namely choosing the appropriate behaviour and gaining social approval, respectively [18]. It has also been argued that injunctive norms facilitate prosocial behaviour while descriptive norms are more relevant to personal benefits [18]. While our results are somewhat consistent with this theory, we would agree with [25] that descriptive norms regarding social behaviour also predict the likely social approval or disapproval one would encounter if deviating from the norm. Thus, descriptive norms may also be expected to motivate prosocial behaviour via similar mechanisms (i.e. to gain social approval/avoid social disapproval) as injunctive norms. Specifically, we would expect individuals to experience more social disapproval if they violate a descriptive social norm, for example by contributing less than most others in a social interaction or by performing antisocial behaviour such as littering in a clean area. Some evidence supports this idea: individuals who violate the established norm of behaviour by either under-contributing, or over-contributing to a public good are more likely to be shunned and punished by their group members [44], [45] and individuals apparently take steps to avoid norm deviance in the context of cooperation [13]. In future studies, it might be useful to ask players whether the extent to which they would disapprove of a low donation (or approve of a high donation) from Player 1 varies according to the descriptive norm information presented.

Some previous work has shown not only that descriptive norms are more effective motivators of the desired behaviour but that injunctive norms may have a counter-productive effect and make individuals more inclined to do the opposite of the behaviour that is socially demanded [22]. In our experiment, we modelled giving nothing to Player 2 as behaving counter to any of the prosocial norms and asked what factors predicted whether Player 1 would give nothing at all to Player 2. We didn’t find any effect of norm type (descriptive versus injunctive) on the propensity of Player 1 to give nothing to Player 2. We were also interested in testing the ‘what the hell’ hypothesis, which has been demonstrated previously in the context of dishonest behaviour. In the honesty experiment, players were shown a screen with a distribution of dots and asked to judge which half of the screen had more dots. The players were given an incentive to respond dishonestly because reporting that the right hand side of the screen had more dots yielded a payoff of $0.05 compared with a payoff of half a cent for the left hand side. Compared to a control situation where each side yields equivalent payoffs, players were more likely to cheat at low levels when they could gain financially from doing so. Moreover, once players had cheated a threshold number of times, they typically switched to an ‘always cheat’ strategy [46]. These effects have been explained in terms of an inability to reconcile the low levels of honesty with a positive self-image and so to instead focus on maximising financial gains [28]. In our experiment, Player 1 might have been more likely to give nothing at all to Player 2 when a high target amount was stated due to a ‘what the hell’ effect. Specifically, specifying a high target amount may be more likely to make Player 1 feel that behaviour consistent with maintaining a positive self-image is too costly and so instead focus on maximising financial gains. In contrast to the predictions of this theory, we did not find that specifying a high target amount ($0.50) was associated with an increased probability to give nothing to Player 2, although it may be the case that specifying even higher target amounts would result in a ‘what the hell’ effect.

To conclude, we have shown that injunctive norms are effective at motivating increasing donations in an anonymous online Dictator Game, whereas descriptive norms did not appear to affect dictator donations. As expected, players were less likely to comply with the norm when doing so involved greater costs ($0.50 compared to $0.20). Nevertheless, higher target amounts did result in increased mean donations. Our data suggest injunctive norms might be used to great effect to motivate prosocial behaviour in real-world settings, for example by museums when soliciting donations from visitors. Future work should explore whether the source of the injunctive norm and the likely beneficiary of the behaviour have an effect on prosocial behaviour.

## Supporting Information

S1 File **Supplementary online material including game instructions, subject information, additional analyses and R code.**
(DOC)

S2 File **Supplementary online material containing data used for analysis.**
(XLSX)
