# Supplementary material for: Dictator Game Giving: The Importance of Descriptive versus Injunctive Norms
Source: PLoS One. 2014 Dec 10;9(12):e113826. doi: 10.1371/journal.pone.0113826 (PMC4262257; doi:10.1371/journal.pone.0113826)
Supplement: S1 File — Supplementary online material including game instructions, subject information, additional analyses and R code. (DOC) [file pone.0113826.s001.doc]

**Supporting Information S1**

**The importance of descriptive versus injunctive norms in promoting cooperative behaviour in an online Dictator Game.**

**N.J. Raihani and K. McAuliffe**

**Table of contents**

|  |  |  |
| --- | --- | --- |
| 1 | Game instructions | p. 2 |
| 2. | Subject information (Table S1) | p. 3 |
| 3. | Analyses of control manipulations from Experiments 1 and 2 | p. 4 |
| 4. | R code for analyses | p. 5 |

**Text transcription of example game instructions received by players, including comprehension questions.**

Screen 1: Please enter your worker ID. This is needed to ensure you get your bonus. If you don't know your Worker ID you can find it out by opening the following page in a new window: https://www.mturk.com/mturk/dashboard

Screen 2: ***GAME INSTRUCTIONS*** You are Player 1. You have been allocated a bonus of $1.00. You can choose how much of this bonus to give to Player 2. Your worker ID and Player 2's worker ID will remain anonymous. Player 2 WILL see these game instructions.

Screen 3: ***ADDITIONAL INFORMATION*** Most Player 1s give $0.20 or more to Player 2. Player 2 WILL see this additional information.

Screen 4: Player 2 will see the game instructions and the additional information.

Screen 5: Please answer these questions correctly to ensure your HIT is accepted. A. In this game, most people: give something to Player 2/keep all the money for themselves.

Screen 6: B Will Player 2 also see the additional information? Yes/No

Screen 7: Well done - you got all the questions right! Ready to play the game? Yes

Screen 8: Please indicate your choice below. You will keep [$1.00 - $0.00]. Player 2 will get [$0.00 - $1.00].

**Table S1**. Information on mean values (where appropriate) and sample sizes for the explanatory terms used in the statistical models, as well as demographic information on education and income levels.

| **Parameter** | **Individuals allocated to role of Player 1 (n = 1,200)** |
| --- | --- |
| Age | Mean = 30 ± 0.3  Range = 18 - 71 |
| Education Level (n) | School = 134  Some University = 408  University Degree = 482  Graduate Degree = 121  Did not specify = 37 |
| Gender (n) | Females = 509  Males = 644  Did not specify = 29 |
|  |  |
| Annual Income (n) | Less than $12,500 = 267  $12,500 - $24,999 = 201  $25,000 - $37,499 = 245  $37,500 - $49,999 = 131  $50,000 - $62,499 = 99  $62,500 - $74,999 = 69  $75,000 or more = 101  Did not disclose = 69 |

**R code used to generate general linear models**

**will update this**

library(MuMIn)

library(arm)

Tables 1 - 3

Models to investigate how norm information (descriptive, injunctive, control) affects tendency to give target amount ($0.20 or $0.50, respectively).

anal<-read.csv("analysis.csv", header=T)

names(anal)

library(arm)

library(lme4)

library(MuMIn)

trt<-subset(anal, Target=="$0.20") #changed to "$0.50" for Table 2.

trt_a<-subset(trt, Gender!="*")

trt_b<-subset(trt_a, Age!="0")

global.model<-glm(bin_complied~Treat+Gender+Age, family="binomial", data=trt_b)

stdz.model<-standardize(global.model, standardize.y=FALSE)

summary(stdz.model)

model.set<-dredge(stdz.model, REML=FALSE)

summary(model.set)

top.models<-get.models(model.set, subset=delta<2)

a<-model.avg(top.models, adjusted=FALSE, revised.var=TRUE)

summary(a)

list(a)

confint(model.avg(top.models))

importance(model.avg(top.models))

avg.model(model.avg(top.models))

Tables 4 & 5

Model to investigate factors affecting mean donation made by Player 1 to Player 2.

trt_a<-subset(anal, Gender!="*")

trt_b<-subset(trt_a, Age!="0")

trt_c<-subset(trt_b, Treat!="control")

global.model<-glm(Given~Treat+Amount+Treat:Amount+Gender+Age, data=trt_c)

stdz.model<-standardize(global.model, standardize.y=FALSE)

summary(stdz.model)

model.set<-dredge(stdz.model, REML=FALSE)

summary(model.set)

top.models<-get.models(model.set, subset=delta<2)

a<-model.avg(top.models, adjusted=FALSE, revised.var=TRUE)

summary(a)

list(a)

confint(model.avg(top.models))

importance(model.avg(top.models))

avg.model(model.avg(top.models))

Tables 6 & 7

Model to investigate whether specifying a high target amount increases likelihood of Player 1 giving nothing to Player 2.

trt_a<-subset(anal, Gender!="*")

trt_b<-subset(trt_a, Age!="0")

trt_c<-subset(trt_b, Treat!="control")

global.model<-glm(bin_nothing~Treat+Amount+Treat:Amount+Gender+Age, family="binomial", data=trt_b)

stdz.model<-standardize(global.model, standardize.y=FALSE)

summary(stdz.model)

model.set<-dredge(stdz.model, REML=FALSE)

summary(model.set)

top.models<-get.models(model.set, subset=delta<2)

a<-model.avg(top.models, adjusted=FALSE, revised.var=TRUE)

summary(a)

list(a)

confint(model.avg(top.models))

importance(model.avg(top.models))

avg.model(model.avg(top.models))
